# Advances in clinical neuro-oncology research on integrin PET imaging

**DOI:** 10.1186/s41824-025-00270-8

**Published:** 2025-09-29

**Authors:** Dylan Henssen, Siem Herings, Osama Sabri, Swen Hesse, Anja van der Kolk, Anne Arens, Martin Gotthardt

**Affiliations:** 1 Department of Medical Imaging, Radboud University Medical Center, Nijmegen, the Netherlands; 2 Department of Nuclear Medicine, University Hospital Leipzig, Leipzig, Germany

**Keywords:** Angiogenesis, Integrins, RGD, Neuro-oncology, Glioma, Brain metastasis

## Abstract

**Background:**

Angiogenesis plays a pivotal role in the progression of neuro-oncological diseases, mediated by integrin receptors on endothelial and tumor cells. Radiolabeled RGD peptides, targeting integrins such as αvβ_3_, offer potential as imaging tracers for diagnosing and monitoring these diseases. This review evaluates the effectiveness and reliability of RGD-containing peptides for PET imaging in neuro-oncology, focusing on diagnostic performance, tumor delineation, and treatment response evaluation.

**Methods:**

A systematic literature search was conducted in PubMed, EMBASE, and Cochrane Library until November 2024, identifying relevant studies using RGD-based tracers in neuro-oncological imaging. Data on patient demographics, tumor types, imaging protocols, tracer characteristics, and outcomes were extracted. Methodological quality was assessed using the QUADAS-2 tool.

**Results:**

Eight studies, encompassing 112 patients with primary and secondary brain tumors, were included. All studies utilized αvβ_3_ integrin expression-targeting RGD PET tracers. Compared to [^18^F]FDG PET, RGD-targeted imaging demonstrated superior tumor-to-background ratios, enabling better detection of neuro-oncological lesions. Only a limited number of studies included histopathological validation, which revealed a strong correlation between integrin expression and tracer uptake. RGD-based imaging also predicted treatment response to chemoradiotherapy and bevacizumab, with significant SUVmax reductions linked to better prognoses. No adverse events related to radiotracers were reported. However, since RGD PET tracers do not cross the blood-brain barrier, the extent to which nonspecific accumulation occurs due to blood-brain barrier disruption in neuro-oncological disease remains partially elusive.

**Conclusion:**

RGD PET imaging is a promising tool for neuro-oncology, providing enhanced diagnostic accuracy and valuable prognostic insights. Future research should focus on integrating molecular imaging findings into personalized treatment strategies and exploring novel RGD tracers for broader clinical applications.

**Supplementary Information:**

The online version contains supplementary material available at 10.1186/s41824-025-00270-8.

## Introduction

Angiogenesis is a complex biological process involved in various physiological and pathological conditions, ranging from wound healing to cancer growth (Folkman 1995; Cao et al. 2011). Being one of the key processes in oncological diseases, angiogenesis could potentially be used for diagnosis and treatment (Folkman 1995). Although angiogenesis is a highly orchestrated process mediated by a plethora of proteins and receptors, one of the major classes of driving receptors concerns the integrins. Integrins are heterodimeric transmembrane glycoproteins that are overly expressed on activated endothelial cells as well as on proliferating tumor cells. They consist of non-covalently associated α and β subunits and eight of the twenty-four integrin heterodimers found in humans recognize RGD (Arg-Gly-Asp) containing peptides(Plow et al. 2000). Although the different subtypes of integrins interact differently with RGD, stimulation of integrins generally leads to tumor growth, tumor invasion and metastasis. First, RGD peptides stimulate the integrins found in the membranes of endothelial cells leading to endothelial cell migration and endothelial cell invasion which in turn drives angiogenesis of tumors (Hynes 2002). Second, stimulation of integrins found in the membranes of tumor cells will promote tumor cell migration and tumor cell invasion. Therefore, integrins are essential for the development of (recurrent) oncological disease and have thus become an interesting substrate of oncological molecular imaging (Xiao and Xin 2022).

The most commonly investigated integrin receptors in the arena of molecular imaging research are the αvβ_3_-, α_5_β_1_- and αvβ_6_ integrins. RGD-containing peptides which have shown to bind to one or more of these integrins concern the cyclic RGD pentapeptides c(RGDyK), c(RGDfK) and c(RGDfV)(Debordeaux et al. 2018). These can be radiolabeled for molecular imaging purposes in different oncological diseases. Neoplasms which show the highest expression of integrins include adult diffuse type gliomas, melanomas, lung- and breast cancer. Together, these oncological entities comprise the vast majority of lesions that account for primary and secondary neuro-oncological disease. Primary brain tumors are characterized according to the World Health Organization 2021 classification (Louis et al. 2021). The largest group consists of adult diffuse gliomas, which are classified as either astrocytic (*IDH*_mut_ 1p/19q intact) or oligodendroglial (*IDH*_mut_ 1p/19q codeleted) based on molecular profiling (Louis et al. 2021). The most common and most fatal form of primary brain tumors is glioblastoma which has an astrocytic origin, has a poor overall survival estimated at 5% at 5 years after diagnosis (Price et al. 2024). Secondary brain tumors, on the other hand, consist of a broad variety of brain metastases and are also associated with a poor overall survival. For all tumor types, overall survival rates are estimated at 2.5% at 5 years after the diagnosis (Hall et al. 2000; Long et al. 2023; Salari et al. 2024). It is believed that improved molecular imaging strategies of both primary and secondary neuro-oncological disease will help to improve overall survival as sophisticated imaging techniques allow us to understand pathophysiological and biological mechanisms which drive tumor genesis and tumor recurrence, before, during and after systemic treatments.

First imaging experiments with healthy volunteers showed that there was no uptake of different RGD-containing peptides in the brain parenchyma, indicating these substances cannot cross the blood brain barrier. Due to the disruption of the blood brain barrier in neuro-oncological disease RGD-containing peptides are able to enter the brain at these local disruptions and bind to integrin receptors present in the neuro-oncological tissue. Theoretically this implies that imaging using RGD-containing peptides provide visualization of neuro-oncological lesions with very little background uptake and thus a high signal-to-background ratio (Yu et al. 2015).

This review will focus on how the integration of findings from existing studies contributes to a comprehensive understanding of the diagnostic performance of RGD-containing tracers, their specificity in binding to integrin receptors, the potential impact on delineating tumor boundaries and the evaluation of therapeutic response.

## Materials and methods

### Literature search and screening

A systematic literature search was performed in PubMed, EMBASE and the Cochrane Library. The search terms included the following: “Glioblastoma”, “Glioma”, “Brain Neoplasms/primary” “Brain metastasis”, “Brain Neoplasms/secondary”, “Positron Emission Tomography Computed Tomography”, “Integrin alphaVbeta3”, “Endothelial Cells”, “Endothelial Cells Metabolism”. The three databases were consulted until November 2024 by a single investigator (D.H.). The complete search string is provided in Table 1.

**Table 1:** Search string per database

Database	Search String
PubMed	(((“Brain Neoplasms“[Mesh]) OR (((brain metasta*[Title/Abstract]) OR (“Brain Neoplasms/secondary“[Mesh]) OR (((glioma[Title/Abstract]) OR (glioblastoma[Title/Abstract])) OR (((“Glioma“[Mesh]) OR “Glioma/diagnostic imaging“[Mesh]) OR ((“Glioblastoma“[Mesh]) OR “Glioblastoma/diagnostic imaging“[Mesh]))))) AND ((((((Positron Emission Tomography Computed Tomography[Title/Abstract]) OR (PET/CT[Title/Abstract])) OR (PET-CT[Title/Abstract])) OR ((“Positron Emission Tomography Computed Tomography“[MAJR]) OR “Positron Emission Tomography Computed Tomography“[MAJR])) AND ((((“Integrin alphaVbeta3/metabolism“[MeSH]) OR “Endothelial Cells/metabolism“[MeSH]) OR (“Integrin alphaVbeta3“[nm])) OR (Integrin alphaVbeta3[Title/Abstract])))))
EMBASE	((Integrin alphaVbeta3.mp. or exp vitronectin receptor/) AND (((glioma or glioblastoma).mp. [mp = title, abstract, heading word, drug trade name, original title, device manufacturer, drug manufacturer, device trade name, keyword heading word, floating subheading word, candidate term word])) OR ((brain metastasis or brain meta*).mp. [mp = title, abstract, heading word, drug trade name, original title, device manufacturer, drug manufacturer, device trade name, keyword heading word, floating subheading word, candidate term word]))) OR ((brain neoplasm*).mp. [mp = title, abstract, heading word, drug trade name, original title, device manufacturer, drug manufacturer, device trade name, keyword heading word, floating subheading word, candidate term word]))) AND ((Positron Emission Tomography Computed Tomography or PET CT).mp. [mp = title, abstract, heading word, drug trade name, original title, device manufacturer, drug manufacturer, device trade name, keyword heading word, floating subheading word, candidate term word]))
Cochrane Library	(brain tumor or brain metastas* or glioma or glioblastoma) AND (positron emission tomography or PET or PET CT or Positron Emission Tomography Computed Tomography) AND (integrin alpha V beta3 or integrin)

The retrieved articles were imported into Rayyan (www.rayyan.ai), a systematic review tool that aids in the blinded screening of publications and detects duplicate findings. After processing the duplicate findings, the remaining titles and abstracts were screened independently by two researchers (D.H., S.H.) blinded to each other’s decisions. During this screening the researchers determined which papers should be included and which should be excluded based on the abstract. Incongruently assessed papers were discussed after which a consensus decision was made. This procedure was followed by a full-text screening of the retrieved articles, utilizing the same setup.

### Extracted data and synthesis of results

The papers included after full-text assessment were analyzed by two investigators independently (D.H. and S.H.). Predefined, standard data-extraction sheets were used and the following data were extracted: (I) Demographic data of studied population, (II) type of tumors included, (III) number of tumors, (IV) utilized radiotracer, (V) dose administered in MBq, (VI) targeted receptor (integrin subtype), (VII) scan time post-injection, (VIII) major findings, and (IX) limitations of the study. A comprehensive overview of the current state of integrin-targeting tracers in neuro-oncology is provided by use of a narrative description of patients, imaging intervention, primary outcome and main limitations of the study.

### Quality assessment

The methodological quality of the included articles was assessed using the QUADAS-2 checklist (Whiting et al. 2011). This checklist was used to assess the risk of bias and concerns regarding applicability in primary diagnostic accuracy studies. Two investigators (D.H. and S.H.) independently assessed the methodological quality of the included articles. Any discrepancies were resolved by discussion.

## Results

**Fig. 1:**
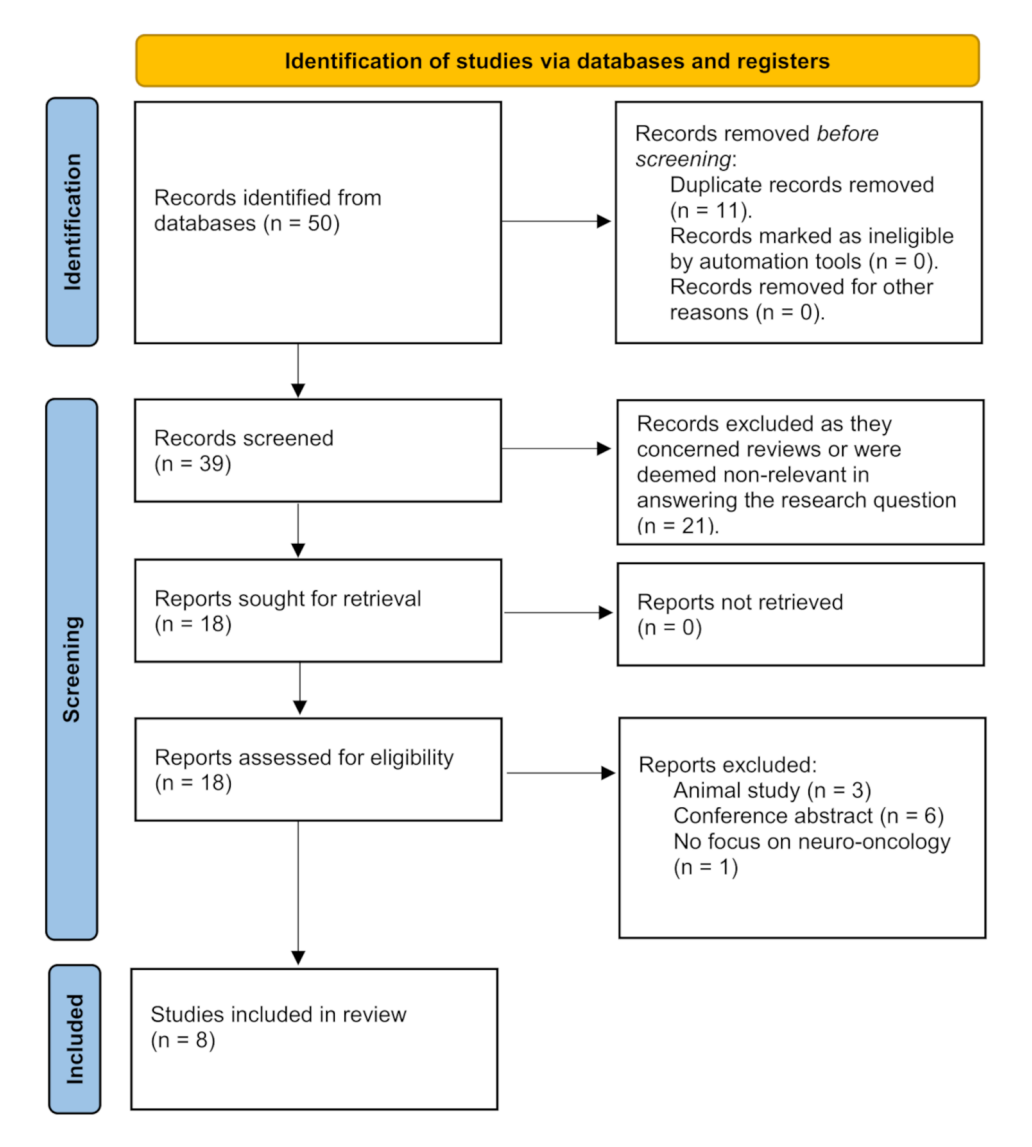
PRISMA 2020 flow diagram for new systematic reviews which included searches of databases and registers only. *Consider, if feasible to do so, reporting the number of records identified from each database or register searched (rather than the total number across all databases/registers). **If automation tools were used, indicate how many records were excluded by a human and how many were excluded by automation tools. *From*: Page MJ, McKenzie JE, Bossuyt PM, Boutron I, Hoffmann TC, Mulrow CD, et al. The PRISMA 2020 statement: an updated guideline for reporting systematic reviews. BMJ 2021;372:n71. doi: 10.1136/bmj.n71

The systematic literature search resulted in 50 articles, of which 11 were duplicates. After removal of these duplicates, the remaining articles (*n* = 39) were screened based on their title and abstract, resulting in the exclusion of 21 articles. The remaining 18 papers were included in the full-text analysis, which led to the exclusion of 10 more articles (animal studies *n* = 3; conference abstracts *n* = 6; studies not focusing on neuro-oncological disease *n* = 1), resulting in 8 articles included in this review (Fig. 1).

In total, 112 patients with primary brain tumors (*n* = 82 lesions) or secondary brain tumors (*n* = 49 lesions) were included in this systematic literature review. One study included five healthy controls (Yu et al. 2015). All included studies used integrin imaging by focusing on the αvβ_3_ integrin subtype. Five studies used PET-CT imaging methods(Yu et al. 2015; Kang et al. 2016; Li et al. 2014, 2018; Zhang et al. 2016), whereas one study used a radiopharmaceutical compound (260-370MBq of [^99m^Tc]IDA-D-[c(RGDfK)]2) that was used for SPECT-CT imaging(Song et al. 2017). The PET imaging studies used different radiotracers: [^18^F]FPPRGD_2_ (Iagaru et al. 2015), [^18^F]Galacto-RGD (Schnell et al. 2009), [^68^Ga](B)NOTA-PRGD2 (Li et al. 2014, 2018), NOTA-E[PEG4-c(RGDfk)]2 (denoted as Alfatide II) which was coupled to either [^18^F] (Yu et al. 2015) or [^68^Ga] (Kang et al. 2016) and [^18^F]ALF-NOTA-PRGD2 (Zhang et al. 2016). An overview of the applied PET-CT imaging protocol per article can be found in Table 2.

**Table 2:** Overview of the included studies

Author (year) (ref)	Participants	Types of tumors		Targeted receptor, tracer and dose	Molecular imaging protocol(s)	Major findings	Secondary findings
		Primary brain tumors	Secondary brain tumors				
Schnell et al. (2009)	12 patients (5 M; mean age: 55.3y)	Glioblastoma (*n* = 4); Recurrent glioblastoma (*n* = 7)	Metastasis from adenocarcinoma of the lung (*n* = 1)	αvβ3, [^18^F]Galacto-RGD, 105–192 MBq	Imaging was performed 40 min post-injection using a Biograph 64 TruePoint TrueV PET-CT (Siemens, Knoxville, TN, USA) with a single bed position for 20 min.	GBM showed significant but heterogeneous tracer uptake, peaking in highly proliferative and infiltrative tumor regions.	In highly proliferative glial tumor regions, tracer uptake (SUVs) on [^18^F]Galacto-RGD PET correlated with immunohistochemical αvβ3 integrin expression in corresponding tumor samples.
Li et al. (2014)	10 patients (10 M; mean age: 43y)	DNET (*n* = 1) Neuronal-glial (*n* = 1) Oligodendroglioma/DNET mixed (*n* = 1) Astrocytoma (*n* = 3) Anaplastic oligoastocytoma (*n* = 2) Glioblastoma (*n* = 5)	N/A	αvβ3, [^68^Ga]BNOTA-PRGD2, 1.85 MBq/kg bodyweight	Imaging was performed 30 min post-injection on a Biograph 64 TruePoint TrueV PET-CT system (Siemens Medical Solutions, Erlangen, Germany) with a single bed position for 10 min.	This prospective clinical study demonstrated that [^68^Ga]BNOTA-PRGD2 PET-CT specifically identifies and assesses glioma neovasculature and tumor cells in glioma patients. [^68^Ga]BNOTA-PRGD2 PET-CT showed higher sensitivity than [^18^F]FDG due to its lack of affinity for normal brain tissue	The TBRmax values of both [^18^F]FDG and [^68^Ga]BNOTA-PRGD2 PET-CT correlated significantly with glioma grading. However, [^18^F]FDG PET-CT could not effectively differentiate low-grade from high-grade gliomas due to overlapping values. In contrast, [68Ga]BNOTA-PRGD2 PET-CT provided superior differentiation. Integrin αvβ3 expression levels in glioma cells corresponded to the histopathological WHO grading.
Iagaru et al. (2015)	17 patients (8 M; mean age: 50y)	Recurrent glioblastoma (*n* = 17)	N/A	αvβ3, [^18^F](PEG3-E[cRGDyk]2 ([^18^F]FPPRGD_2_) 1.85 MBq/kg bodyweight	Brain imaging was performed directly after injection on a GE Discovery 600 or a GE Discovery 690 PET-CT scanner (GE Healthcare, Chicago, Illinois, USA) with a single bed position for 45 min. A total body PET-CT scan was obtained 60 min and 120 min post-injection.	Of the 17 patients, 15 (88%) had recurrent glioblastoma identified on [^18^F]FPPRGD2 PET, while [^18^F]FDG PET detected recurrence in 13 (76%) patients. Two patients (12%) showed no recurrence.	[^18^F]FPPRGD2 is a safe PET radiopharmaceutical with increased uptake in recurrent glioblastoma lesions. Patients showing a decrease of less than 15% in SUVmax and angiogenesis volume on [^18^F]FPPRGD2 PET one week after bevacizumab administration tended to have a very poor prognosis. In contrast, those with a decrease of more than 50% had a better prognosis.
Yu et al. (2015)	9 patients (5 M; mean age: 55y) 5 healthy controls (3 M; mean age: 35y)	N/A	Lung (*n* = 5) Ovarian (*n* = 1) Endometrial (*n* = 1), Gastric (*n* = 1), CUP (*n* = 1)	αvβ3, [^18^F]Alfatide II, 257 ± 48 MBq	Total-body imaging was performed at 5, 10, and 15 min post-injection (40 s/bed position) and at 30, 45, and 60 min post-injection (2 min/bed position) using a Biograph 64 PET-CT (Siemens, Nuremberg, Germany).	All 20 metastatic brain lesions were clearly visualized on [^18^F]Alfatide II PET due to its high TBR values. In contrast, only 10 lesions were detectable on [^18^F]FDG PET, which showed significantly lower TBR values.	[^18^F]Alfatide II was safe and well tolerated in all five healthy volunteers with no adverse events. In healthy volunteers, tracer uptake in the normal brain was very low at 60 min post-injection, indicating that [^18^F]Alfatide II does not cross the blood-brain barrier. A large variation in SUVmax values was observed, likely reflecting differences in integrin expression across lesions of different origins and indicating substantial inter- and intra-individual variability in αvβ3 expression among cancer patients.
Kang et al. (2016)	21 patients (14 M; mean age: N/R)	N/A	NSCLC (*n* = 21)	αvβ3, [^68^Ga]Alfatide II, 1.85 MBq/kg bodyweight	Total body imaging was performed 60 min post-injection on a Biograph 64 PET-CT scanner (Siemens Medical Solutions, Nuremberg, Germany).	[^68^Ga]Alfatide II is better suited for detecting brain metastases, while [^18^F]FDG is more effective for identifying liver and early-stage bone metastases.	[^68^Ga]Alfatide II may be more effective for brain metastasis imaging due to its inability to cross the blood-brain barrier, resulting in a clear background of normal brain tissue.
Zhang et al. (2016)	25 patients (15 M; mean age: 49.5y)	Glioblastoma (total *n* = 25)	N/A	αvβ3, [^18^F]ALF-NOTA-PRGD2, 224.56 ± 38.2 MBq	Brain imaging was performed 50 min post-injection on a Discovery LS PET-CT (GE Healthcare, Chicago, Illinois, USA)	[^18^F]ALF-NOTA-PRGD2 demonstrates excellent in vitro serum stability and in vivo tumor imaging capability. [^18^F]ALF-NOTA-PRGD2 PET-CT parameters can predict sensitivity to concurrent chemoradiotherapy as early as the third week, when the radiation dose reaches 30 Gy.	The upregulated expression of αvβ3 in injured normal brain tissue following surgical resection complicated the interpretation of pretreatment PET parameters in the operation area and residual lesion. ROC curve analysis showed that the predictive ability of [^18^F]AlF-NOTA-PRGD2 PET-CT parameters for short-term outcomes was superior to MRI volumetric parameters. Angiogenesis imaging with RGD PET may reflect the treatment sensitivity of glioblastoma.
Song et al. (2017)	7 patients (3 M; mean age: 64.7y)	Glioblastoma (*n* = 4) Anaplastic astrocytoma (*n* = 1) Meningioma (*n* = 1)	Metastasis of adenocarcinoma of the lung (*n* = 1)	αvβ3, [^99m^Tc]IDA-D-[c(RGDfK)]2, 260–370 MBq	Brain imaging was performed 30 min post-injection on a triple-head TRIAD system (Trionix Research Laboratory, Twinsburg, OH). SPECT acquisitions were performed using a 360° circular orbit detector rotation for 30 min.	Due to high TBR values, integrin αvβ3 overexpression in primary and secondary brain tumors can be clearly identified and localized. The diagnostic value of integrin αvβ3-targeted imaging for brain tumors can be enhanced with [^99m^Tc]IDA-D-[c(RGDfK)]2 SPECT-CT, which demonstrated the ability to detect activated angiogenesis in solid tumors in this study.	Integrin αvβ3 is now recognized to have both positive and negative regulatory roles in angiogenesis, depending on the pathology. Therefore, these results should be interpreted with caution. The injection of [^99m^Tc]IDA-D-[c(RGDfK)]2 was safe and well tolerated, with no clinically significant safety concerns. [^99m^Tc]IDA-D-[c(RGDfK)]2 SPECT-CT may also enable the prediction and monitoring of the clinical efficacy of antiangiogenic agents in malignant tumors.
Li et al. (2018)	21 patients (14 M; mean age 48.5y)	Glioma grade 3 (*n* = 7), Glioblastoma (*n* = 9), Meningioma (*n* = 5)	N/A	αvβ3, [^68^Ga]NOTA-PRGD2, 1.85 MBq/kg bodyweight	Brain imaging was performed 30 min post-injection on an unspecified PET-CT system.	This study identified a specific imaging pattern in meningioma with adjacent brain edema, characterized by low [^18^F]FDG uptake but intense [68Ga]NOTA-PRGD2 accumulation. In contrast, high-grade glioma showed moderate to high [^18^F]FDG uptake but only moderate [68Ga]NOTA-PRGD2 accumulation.	Positive PET imaging with [^68^Ga]NOTA-PRGD2 in these uncommon meningiomas may be attributed to their typically abundant vasculature.

For comparison, four studies used 2-[fluorine-18]fluoro-2-deoxy-d-glucose ([^18^F]FDG) PET imaging of the brain for detection of brain lesions (Yu et al. 2015; Kang et al. 2016; Li et al. 2014; Iagaru et al. 2015). All studies described that RGD-targeted PET imaging was superior to [^18^F]FDG PET imaging of the brain for the detection of brain lesions (both primary and secondary brain tumors) due to the favorable tumor-to-background ratio (TBR). Nevertheless, the study of Li et al. Illustrated that high grade gliomas showed only a moderate [^68^Ga]NOTA-PRGD2 accumulation (Li et al. 2018). In the setting of secondary brain tumors, a large variance of the values of SUVmax was described. This was believed to reflect the difference of integrin expression in lesions of different origins and indicate great inter- and intra-individual variation of αvβ_3_ expression in cancer patients (Yu et al. 2015).

Immunohistochemical αvβ_3_ integrin expression of corresponding tumor samples was found to be highly correlated with the level of tracer uptake according to the study of Schnell et al. (Schnell et al. 2009). None of the other included studies performed histopathological validation of the αvβ_3_ integrin expression and the tracer uptake. The degree of tracer accumulation (i.e., [^68^Ga]BNOTA-PRGD2), however, was also found to be positively correlated to the histopathological assessment of WHO grade (Li et al. 2014).

Furthermore, it has been suggested by two articles that RGD-targeted PET (i.e., [^18^F]FPPRGD2 and [^18^F]ALF-NOTA-PRGD2, respectively), could be used to determine treatment sensitivity of glioblastoma for bevacizumab (Iagaru et al. 2015) and concurrent chemoradiotherapy (Zhang et al. 2016). More specifically, when using [^18^F]FPPRGD2 PET imaging, it was found that participants with a decrease of less than 15% of the maximum standardized uptake value (SUVmax) and reduction of uptake volume (reflecting the angiogenesis volume) after one week of bevacizumab administration tended to have a very poor prognosis. On the other hand, patients showing a decrease of at least 50% in SUVmax and angiogenesis volume one week after bevacizumab administration were found to have a better prognosis. Interestingly, when changes in SUVmax and angiogenesis volumes were discordant, the changes in angiogenesis volume at one week follow-up appeared to be more predictive of the outcome than the changes in SUVmax (Iagaru et al. 2015). When using [^18^F]ALF-NOTA-PRGD2 PET-CT, it was described that sensitivity to concurrent chemoradiotherapy (at least 30 Gy) could be predicted as early as the third week after treatment. Parameters derived from [^18^F]ALF-NOTA-PRGD2 PET-CT imaging during treatment were the SUVmax of the tumor and TBR values. According to receiver operator characteristics (ROC) curve analyses, the parameter with the highest predictive power was the SUVmax of the tumor. SUVmax of the tumor showed an area under the curve (AUC) of 0.833 to predict the short-term outcome of concurrent chemoradiotherapy. When a SUVmax threshold of 1.35 was applied, the sensitivity, specificity, and accuracy were 83.3%, 88.9%, and 85.7%, respectively. The TBR values provided an AUC of 0.769 and a threshold at 19.3 provided a sensitivity, specificity, and accuracy of 75.0%, 88.9%, and 81.0%, respectively (Zhang et al. 2016).

Finally, two papers discussed that RGD-targeted PET imaging could be used in the post-treatment setting as well. In the first study, Schnell et al. discussed that tracer dynamics of [^18^F]Galacto-RGD did not differ between recurrent glioblastoma lesions and de novo glioblastoma lesions (Schnell et al. 2009). When using [^18^F]FPPRGD_2_ PET-CT imaging to discern tumor recurrence from treatment-related abnormalities, Iagaru et al. reported that of the 17 patients, 88% had recurrent glioblastoma identified on [^18^F]FPPRGD_2_ PET, whereas [^18^F]FDG PET enabled identification of recurrence in 76% of patients. In one patient (6%) with recurrent glioblastoma, abnormalities were detected only on [^18^F]FPPRGD_2_ PET, whereas all other patients also showed abnormalities on MRI. Two patients (12%) had no signs of recurrent glioblastoma on PET or MRI (Iagaru et al. 2015).

None of the included studies reported on adverse events after the injection of radiotracer for either PET-CT or SPECT-CT imaging (Yu et al. 2015; Kang et al. 2016; Li et al. 2014, 2018; Zhang et al. 2016; Song et al. 2017; Iagaru et al. 2015; Schnell et al. 2009), although this was specifically mentioned as a secondary outcome in only three studies (Yu et al. 2015; Song et al. 2017; Iagaru et al. 2015).

Quality assessment of the papers revealed that for most elements, the risk of bias of the included studies was determined to be low (Table 3). It was unclear for four of the five studies that used a reference standard (i.e., [^18^F]FDG PET) whether the index test and reference standard were assessed in a blinded setting. Only the article of Iagaru et al. detailed that the index test (i.e., [^18^F]FPPRGD_2_ PET-CT) outcomes were assessed without knowledge of the outcome of the reference standard (i.e., [^18^F]FDG PET-CT images and brain MRI data) and vice versa (Iagaru et al. 2015).

**Table 3:** QUADAS-2 based evaluation of the risk of bias and applicability of primary diagnostic accuracy studies

Author (year) ^ref^	Patient Selection	Index Test	Reference Standard	Flow and Timing
Schnell et al. (2009)	Low	Unclear	Low	Low
Li et al. (2014)	Low	Unclear	Low	Low
Iagaru et al. (2015)	Low	Low	Low	Low
Yu et al. (2015)	Low	Unclear	Low	Low
Kang et al. (2016)[14]	Low	Unclear	Low	Low
Zhang et al. (2016)	Low	Unclear	Low	Low
Song et al. (2017)[18]	Low	Unclear	Low	Low
Li et al. (2018)	Low	Unclear	Low	Low

## Discussion

This systematic review reports on the advantages and challenges of molecular imaging techniques aimed at angiogenesis in neuro-oncological disease by use of radiolabeled RGD peptides. Despite substantial differences in imaging protocols, overall it can be concluded that RGD-targeted PET-CT and SPECT-CT imaging targeting the αvβ_3_ integrin subtype demonstrates superiority over [^18^F]FDG PET-CT in detecting primary and secondary brain tumor lesions due to its higher TBR values, and shows potential for predicting treatment response and post-therapeutic evaluation. One study reported a strong correlation between tracer binding (evaluated with SUVmax) and integrin expression after histopathological evaluation. None of the studies reported adverse events, underscoring its clinical applicability. The heterogeneity in used RGD-based tracers might, at least in part, explain the difference in tracer accumulation between studies. It has been shown that the linker group has little impact on the αvβ_3_ binding affinity of cyclic RGD dimers. The αvβ_3_ binding affinity of NOTA-4P-RGD3 was found to be almost identical to that of NOTA-Galacto-RGD2 and NOTA-I2P-RGD2, despite the differences in peptide multiplicity. The αvβ_3_ binding affinity of DOTA-3P-RGK2, on the other hand, was found to be at least twenty times lower than those of NOTA-Galacto-RGD2, NOTA-I2P-RGD2, NOTA-4P-RGD3, suggesting that αvβ_3_-binding of these RGD-compounds is highly specific (Zhao et al. 2019).

### Specific binding vs. non-specific accumulation

A variety of RGD-based tracers targeting the αvβ_3_ integrin subtype have been reviewed in this study and the majority of studies showed a high tracer accumulation within gliomas, brain metastases and meningioma. Since RGD-radiolabeled tracers cannot cross the intact blood-brain barrier (Yu et al. 2015), it can be assumed that tracer accumulation is at least in part the result of non-specific accumulation due to more pronounced leakage of the tracer. This leakage can be caused by the severely disintegrated blood-brain barrier in more aggressive gliomas or may be the result from leaking tumor micro-vessels. However, in the study of Li et al. (Li et al. 2014), it can be appreciated that regions with contrast-enhancement on MRI do not necessarily overlap with regions of high tracer accumulation. Since contrast-enhancement is a marker of blood-brain barrier disruption, this observation is an argument against solely non-specific leakage of the tracer. Furthermore, another study by Li et al. observed only a moderate [^68^Ga]NOTA-PRGD2 accumulation in high grade gliomas (Li et al. 2018). This also forms an argument against non-specific tracer accumulation. Immunohistochemical αvβ_3_ integrin expression of corresponding tumor samples was found to be highly correlated with the level of tracer uptake according to the study of Schnell et al. (Schnell et al. 2009). Unfortunately, no other studies are known that performed histopathological validation of the αvβ_3_ integrin expression and tracer uptake. Future studies could help to solve the question with regard to the binding potential of RGD-based tracers to the αvβ_3_ integrin subtype in adult diffuse glioma. For example, a dual-tracer imaging protocol study could help to discern specific from non-specific binding. On the first day, a perfusion PET-tracer (e.g., [^15^O]H_2_O, [^13^N]Ammonia, and ^82^Rb) is administered to assess perfusion and leakage of the tracer through the disintegrated blood brain barrier. The second PET-assessment will be carried out using a PET-tracer targeting αvβ_3_ integrin. By comparing these data within-subject, the component of specific vs. non-specific binding will become apparent.

### Alternative PET agents targeting angiogenesis

The current review focuses on RGD-based imaging tracers for the visualization of angiogenesis, a process much needed for neuro-oncological lesions to provide nutrients and other building blocks (Hardee and Zagzag 2012). Tumor cells facilitate this by initiating and upregulating angiogenesis, significantly increasing the number of vessels supplying the tumor. Angiogenesis is, however, a complex process in which tissue cells and their surrounding stroma interact and produce growth factors like vascular endothelial growth factor (VEGF) which attract and stimulate endothelial and mesenchymal cells to form new (micro)vessels (Colman 2011; Nussenbaum and Herman 2010; Palma et al. 2017).

Next to radiolabeled RGD-peptides aimed at the integrins, a variety of other PET tracers have been developed to target angiogenesis and newly formed (micro)vessels. [^68^Ga]PSMA, [^18^F]DCFPyL and [^89^Zr]Df-IAB2M have been described to bind to the prostate-specific membrane antigen (PSMA). PSMA, which is less well-known as glutamate carboxypeptidase 2 is a receptor that is believed to induce angiogenesis in pathological conditions like tumors independently from the presence of VEGF. PSMA is variably expressed on newly formed blood vessels in tumors, while it is not expressed on healthy brain parenchymal cells or normal vessels. As transport over the blood-brain barrier is impossible, the blood-brain barrier needs to be disintegrated and by consequence, radiotracer accumulation depends on the tumor type (Nomura et al. 2014). This is similar to radiolabeled RGD-peptides aimed at the integrins. [^68^Ga]PSMA is used most often because of its wide availability for prostate cancer imaging. [^18^F]DCFPyL and other [^18^F]-coupled radiotracers have similar biological properties, though [^89^Zr]Df-IAB2M is a small part of the PSMA antibody and shows faster clearance, thereby achieving higher TBR values as compared to the other two agents (Matsuda et al. 2018). Although preliminary work showed high TBR values in glioblastoma patients (Brighi et al. 2023; Kunikowska et al. 2018, 2020), a recent multicenter study in which different PSMA tracers were applied showed that there was no correlation between tracer uptake and PSMA receptor density on tumor cells of microvessels (Lith et al. 2023). This forms a strong argument that PSMA tracers show non-specific accumulation in adult diffuse glioma and represent increased permeability of the blood brain barrier, in combination with leaky tumor microvessels (Nussenbaum and Herman 2010; Palma et al. 2017). This hypothesis is strengthened by reports on high uptake in enhancing radiation necrosis and ischemia (Sasikumar et al. 2017; Salas Fragomeni et al. 2018).

Another promising strategy for imaging tumor angiogenesis concerns the targeting of VEGF. VEGFs are a family of mitogenic glycoproteins that promote angiogenesis by the activation of the VEGF receptor via a tyrosine kinase signaling pathway (Jubb and Harris 2010). ^64^Cu-1,4,7-triazacyclononane-1,4,7-triacetic acid-*p*-isothiocyanatobenzyl-bevacizumab-IRDye 800CW (^64^Cu-NOTA-Bev-800CW), [^89^Zr]bevacizumab (Nagengast et al. 2007) and [^89^Zr]ranibizumab (Nagengast et al. 2011) have been proposed as PET tracers to study human VEGF levels. In one study, it has been described that the [^89^Zr]ranibizumab-PET signal is the sum of perfusion of the tracer into the tumor followed by binding to VEGF, and therefore a resultant of changed perfusion, mean vessel density and VEGF expression, reflecting VEGF biodistribution and bioavailability and allowing in vivo insight in overall tumor angiogenesis (Nagengast et al. 2011).

### Possible impact in the clinical setting

In the setting of discerning recurrence from treatment-related abnormalities in post-treatment glioblastoma patients, the Response Assessment in Neuro-Oncology working group has recommended the use of PET imaging (Albert et al. 2016). A variety of PET tracers are available for this purpose and recent meta-analyses investigated the diagnostic accuracy of each. O-(2-[^18^F]fluoroethyl)-L-tyrosine ([^18^F]FET) and [S-methyl-^11^C]methionine ([^11^C]MET) were reportedly the most accurate radiotracers for this clinical purpose (Zwart et al. 2020; Henssen et al. 2023). However, the works of Schnell et al. (Schnell et al. 2009) and Iagaru et al. (Iagaru et al. 2015) indicate that RGD-based PET imaging could be a valuable diagnostic tool for this purpose as well. More research is needed as there are no prospective studies available on this topic. When considering future research directions for the clinical application of RGD-targeted PET imaging, we recommend that researchers prioritize the integration of molecular imaging findings into personalized treatment strategies. This approach has the potential to enhance patient outcomes by tailoring therapies to the unique biological characteristics of individual tumors. Such advancements could facilitate broader utilization in diverse medical contexts, further solidifying the role of RGD-targeted PET imaging in modern precision medicine for neuro-oncology patients. However, as mentioned before, the binding specificity of RGD-tracers should be investigated first. Another relevant topic prior to clinical implementation concerns the large variation in SUVmax values described by Yu et al. (Yu et al. 2015). The authors hypothesized that this large variation probably reflects differences in integrin expression across lesions of different origins and variability in αvβ_3_ expression among cancer patients. For that reason, within subject-normalization could be considered in the clinical setting.

## Conclusion

This review shows that RGD-targeted PET and SPECT imaging could be a promising tool for neuro-oncology as it provides new molecular information on the lesions. However, the specificity of RGD-tracers remains partially elusive and should therefore be studied further before RGD-targeted PET can be implemented in clinical practice. Radiopharmacodynamic, -kinetic and modelling studies in humans are much needed to bring this type of tracer further from bench to bedside in patients with neurooncological disorders.

## Supplementary Information

Below is the link to the electronic supplementary material.

Supplementary Material 1

Supplementary Material 2

## Data Availability

No specific datasets were generated during the current study; all required information on the origin of the data of this review is made available from this manuscript.
