# Supplementary material for: Advances in clinical neuro-oncology research on integrin PET imaging
Source: EJNMMI Rep. 2025 Sep 29;9(1):33. doi: 10.1186/s41824-025-00270-8 (PMC12477096; doi:10.1186/s41824-025-00270-8)
Supplement: Supplementary file 1 — Supplementary Material 1 [file 41824_2025_270_MOESM1_ESM.docx]

Advances in clinical neuro-oncology research on integrin PET imaging

Dylan Henssen^1,2^* [dylan.henssen@radboudumc.nl](mailto:dylan.henssen@radboudumc.nl) / dylan.henssen@medizin.uni-leipzig.de

Siem Herings ^1^* [siem.herings@radboudumc.nl](mailto:siem.herings@radboudumc.nl)

Osama Sabri ^2^ [osama.sabri@medizin.uni-leipzig.de](mailto:osama.sabri@medizin.uni-leipzig.de)

Swen Hesse ^2^ [swen.hesse@medizin.uni-leipzig.de](mailto:swen.hesse@medizin.uni-leipzig.de)

Anja van der Kolk ^1^ [anja.vanderkolk@radboudumc.nl](mailto:anja.vanderkolk@radboudumc.nl)

Anne I.J. Arens ^1^ [anne.arens@radboudumc.nl](mailto:anne.arens@radboudumc.nl)

Martin Gotthardt ^1^ [martin.gotthardt@radboudumc.nl](mailto:martin.gotthardt@radboudumc.nl)

^1^ Department of Medical Imaging, Radboud University Medical Center, Nijmegen, the Netherlands
^2^ Department of Nuclear Medicine, University Hospital Leipzig, Leipzig, Germany
 * Authors contributed equally

Corresponding author:

Dr. Dylan Henssen

[dylan.henssen@radboudumc.nl](mailto:dylan.henssen@radboudumc.nl)

dylan.henssen@medizin.uni-leipzig.de

# Abstract

**Background:** Angiogenesis plays a pivotal role in the progression of neuro-oncological diseases, mediated by integrin receptors on endothelial and tumor cells. Radiolabeled RGD peptides, targeting integrins such as αvβ_3_, offer potential as imaging tracers for diagnosing and monitoring these diseases. This review evaluates the effectiveness and reliability of RGD-containing peptides for PET imaging in neuro-oncology, focusing on diagnostic performance, tumor delineation, and treatment response evaluation.

**Methods**: A systematic literature search was conducted in PubMed, EMBASE, and Cochrane Library until November 2024, identifying relevant studies using RGD-based tracers in neuro-oncological imaging. Data on patient demographics, tumor types, imaging protocols, tracer characteristics, and outcomes were extracted. Methodological quality was assessed using the QUADAS-2 tool.

**Results**: Eight studies, encompassing 112 patients with primary and secondary brain tumors, were included. All studies utilized αvβ_3_ integrin expression-targeting RGD PET tracers. Compared to [^18^F]FDG PET, RGD-targeted imaging demonstrated superior tumor-to-background ratios, enabling better detection of neuro-oncological lesions. Only a limited number of studies included histopathological validation, which revealed a strong correlation between integrin expression and tracer uptake. RGD-based imaging also predicted treatment response to chemoradiotherapy and bevacizumab, with significant SUVmax reductions linked to better prognoses. No adverse events related to radiotracers were reported. However, since RGD PET tracers do not cross the blood-brain barrier, the extent to which nonspecific accumulation occurs due to blood-brain barrier disruption in neuro-oncological disease remains partially elusive.

**Conclusion:** RGD PET imaging is a promising tool for neuro-oncology, providing enhanced diagnostic accuracy and valuable prognostic insights. Future research should focus on integrating molecular imaging findings into personalized treatment strategies and exploring novel RGD tracers for broader clinical applications.

# Introduction

Angiogenesis is a complex biological process involved in various physiological and pathological conditions, ranging from wound healing to cancer growth [1, 2]. Being one of the key processes in oncological diseases, angiogenesis could potentially be used for diagnosis and treatment [1]. Although angiogenesis is a highly orchestrated process mediated by a plethora of proteins and receptors, one of the major classes of driving receptors concerns the integrins. Integrins are heterodimeric transmembrane glycoproteins that are overly expressed on activated endothelial cells as well as on proliferating tumor cells. They consist of non-covalently associated α and β subunits and eight of the twenty-four integrin heterodimers found in humans recognize RGD (Arg-Gly-Asp) containing peptides[3]. Although the different subtypes of integrins interact differently with RGD, stimulation of integrins generally leads to tumor growth, tumor invasion and metastasis. First, RGD peptides stimulate the integrins found in the membranes of endothelial cells leading to endothelial cell migration and endothelial cell invasion which in turn drives angiogenesis of tumors [4]. Second, stimulation of integrins found in the membranes of tumor cells will promote tumor cell migration and tumor cell invasion. Therefore, integrins are essential for the development of (recurrent) oncological disease and have thus become an interesting substrate of oncological molecular imaging [5].

The most commonly investigated integrin receptors in the arena of molecular imaging research are the αvβ_3_-, α_5_β_1_- and αvβ_6_ integrins. RGD-containing peptides which have shown to bind to one or more of these integrins concern the cyclic RGD pentapeptides c(RGDyK), c(RGDfK) and c(RGDfV)[6]. These can be radiolabeled for molecular imaging purposes in different oncological diseases. Neoplasms which show the highest expression of integrins include adult diffuse type gliomas, melanomas, lung- and breast cancer. Together, these oncological entities comprise the vast majority of lesions that account for primary and secondary neuro-oncological disease. Primary brain tumors are characterized according to the World Health Organization 2021 classification [7]. The largest group consists of adult diffuse gliomas, which are classified as either astrocytic (*IDH*_mut_ 1p/19q intact) or oligodendroglial (*IDH*_mut_ 1p/19q codeleted) based on molecular profiling [7]. The most common and most fatal form of primary brain tumors is glioblastoma which has an astrocytic origin, has a poor overall survival estimated at 5% at 5 years after diagnosis [8]. Secondary brain tumors, on the other hand, consist of a broad variety of brain metastases and are also associated with a poor overall survival. For all tumor types, overall survival rates are estimated at 2.5% at 5 years after the diagnosis [9-11]. It is believed that improved molecular imaging strategies of both primary and secondary neuro-oncological disease will help to improve overall survival as sophisticated imaging techniques allow us to understand pathophysiological and biological mechanisms which drive tumor genesis and tumor recurrence, before, during and after systemic treatments.

First imaging experiments with healthy volunteers showed that there was no uptake of different RGD-containing peptides in the brain parenchyma, indicating these substances cannot cross the blood brain barrier. Due to the disruption of the blood brain barrier in neuro-oncological disease RGD-containing peptides are able to enter the brain at these local disruptions and bind to integrin receptors present in the neuro-oncological tissue. Theoretically this implies that imaging using RGD-containing peptides provide visualization of neuro-oncological lesions with very little background uptake and thus a high signal-to-background ratio [12].

This review will focus on how the integration of findings from existing studies contributes to a comprehensive understanding of the diagnostic performance of RGD-containing tracers, their specificity in binding to integrin receptors, the potential impact on delineating tumor boundaries and the evaluation of therapeutic response.

# Materials and Methods

## Literature search and screening

A systematic literature search was performed in PubMed, EMBASE and the Cochrane Library. The search terms included the following: “Glioblastoma”, “Glioma”, "Brain Neoplasms/primary" “Brain metastasis”, "Brain Neoplasms/secondary", “Positron Emission Tomography Computed Tomography”, "Integrin alphaVbeta3", "Endothelial Cells”, “Endothelial Cells Metabolism". The three databases were consulted until November 2024 by a single investigator (D.H.). The complete search string is provided in **Table 1**.

INSERT TABLE 1 HERE

The retrieved articles were imported into Rayyan ([www.rayyan.ai](http://www.rayyan.ai)), a systematic review tool that aids in the blinded screening of publications and detects duplicate findings. After processing the duplicate findings, the remaining titles and abstracts were screened independently by two researchers (D.H., S.H.) blinded to each other’s decisions. During this screening the researchers determined which papers should be included and which should be excluded based on the abstract. Incongruently assessed papers were discussed after which a consensus decision was made. This procedure was followed by a full-text screening of the retrieved articles, utilizing the same setup.

## Extracted data and synthesis of results

The papers included after full-text assessment were analyzed by two investigators independently (D.H. and S.H.). Predefined, standard data-extraction sheets were used and the following data were extracted: I) Demographic data of studied population, II) type of tumors included, III) number of tumors, IV) utilized radiotracer, V) dose administered in MBq, VI) targeted receptor (integrin subtype), VII) scan time post-injection, VIII) major findings, and IX) limitations of the study. A comprehensive overview of the current state of integrin-targeting tracers in neuro-oncology is provided by use of a narrative description of patients, imaging intervention, primary outcome and main limitations of the study.

## Quality assessment

The methodological quality of the included articles was assessed using the QUADAS-2 checklist [13]. This checklist was used to assess the risk of bias and concerns regarding applicability in primary diagnostic accuracy studies. Two investigators (D.H. and S.H.) independently assessed the methodological quality of the included articles. Any discrepancies were resolved by discussion.

# Results

The systematic literature search resulted in 50 articles, of which 11 were duplicates. After removal of these duplicates, the remaining articles (n=39) were screened based on their title and abstract, resulting in the exclusion of 21 articles. The remaining 18 papers were included in the full-text analysis, which led to the exclusion of 10 more articles (animal studies n=3; conference abstracts n=6; studies not focusing on neuro-oncological disease n=1), resulting in 8 articles included in this review (**Figure 1**).

In total, 112 patients with primary brain tumors (n=82 lesions) or secondary brain tumors (n=49 lesions) were included in this systematic literature review. One study included five healthy controls [12]. All included studies used integrin imaging by focusing on the αvβ_3_ integrin subtype. Five studies used PET-CT imaging methods[12, 14-17], whereas one study used a radiopharmaceutical compound (260-370MBq of [^99m^Tc]IDA-D-[c(RGDfK)]2) that was used for SPECT-CT imaging[18]. The PET imaging studies used different radiotracers: [^18^F]FPPRGD_2_ [19], [^18^F]Galacto-RGD [20], [^68^Ga](B)NOTA-PRGD2 [15, 16], NOTA-E[PEG4-c(RGDfk)]2 (denoted as Alfatide II) which was coupled to either [^18^F] [12] or [^68^Ga] [14] and [^18^F]ALF-NOTA-PRGD2 [17]. An overview of the applied PET-CT imaging protocol per article can be found in **Table 2**.

INSERT TABLE 2 HERE

For comparison, four studies used 2-[fluorine-18]fluoro-2-deoxy-d-glucose ([^18^F]FDG) PET imaging of the brain for detection of brain lesions [12, 14, 16, 19]. All studies described that RGD-targeted PET imaging was superior to [^18^F]FDG PET imaging of the brain for the detection of brain lesions (both primary and secondary brain tumors) due to the favorable tumor-to-background ratio (TBR). Nevertheless, the study of Li et al. Illustrated that high grade gliomas showed only a moderate [^68^Ga]NOTA-PRGD2 accumulation [15]. In the setting of secondary brain tumors, a large variance of the values of SUVmax was described. This was believed to reflect the difference of integrin expression in lesions of different origins and indicate great inter- and intra-individual variation of αvβ_3_ expression in cancer patients [12].

Immunohistochemical αvβ_3_ integrin expression of corresponding tumor samples was found to be highly correlated with the level of tracer uptake according to the study of Schnell et al. [20]. None of the other included studies performed histopathological validation of the αvβ_3_ integrin expression and the tracer uptake. The degree of tracer accumulation (i.e., [^68^Ga]BNOTA-PRGD2), however, was also found to be positively correlated to the histopathological assessment of WHO grade [16].

Furthermore, it has been suggested by two articles that RGD-targeted PET (i.e., [^18^F]FPPRGD2 and [^18^F]ALF-NOTA-PRGD2, respectively), could be used to determine treatment sensitivity of glioblastoma for bevacizumab [19] and concurrent chemoradiotherapy [17]. More specifically, when using [^18^F]FPPRGD2 PET imaging, it was found that participants with a decrease of less than 15% of the maximum standardized uptake value (SUVmax) and reduction of uptake volume (reflecting the angiogenesis volume) after one week of bevacizumab administration tended to have a very poor prognosis. On the other hand, patients showing a decrease of at least 50% in SUVmax and angiogenesis volume one week after bevacizumab administration were found to have a better prognosis. Interestingly, when changes in SUVmax and angiogenesis volumes were discordant, the changes in angiogenesis volume at one week follow-up appeared to be more predictive of the outcome than the changes in SUVmax [19]. When using [^18^F]ALF-NOTA-PRGD2 PET-CT, it was described that sensitivity to concurrent chemoradiotherapy (at least 30 Gy) could be predicted as early as the third week after treatment. Parameters derived from [^18^F]ALF-NOTA-PRGD2 PET-CT imaging during treatment were the SUVmax of the tumor and TBR values. According to receiver operator characteristics (ROC) curve analyses, the parameter with the highest predictive power was the SUVmax of the tumor. SUVmax of the tumor showed an area under the curve (AUC) of 0.833 to predict the short-term outcome of concurrent chemoradiotherapy. When a SUVmax threshold of 1.35 was applied, the sensitivity, specificity, and accuracy were 83.3%, 88.9%, and 85.7%, respectively. The TBR values provided an AUC of 0.769 and a threshold at 19.3 provided a sensitivity, specificity, and accuracy of 75.0%, 88.9%, and 81.0%, respectively [17].

Finally, two papers discussed that RGD-targeted PET imaging could be used in the post-treatment setting as well. In the first study, Schnell et al. discussed that tracer dynamics of [^18^F]Galacto-RGD did not differ between recurrent glioblastoma lesions and de novo glioblastoma lesions [20]. When using [^18^F]FPPRGD_2_ PET-CT imaging to discern tumor recurrence from treatment-related abnormalities, Iagaru et al. reported that of the 17 patients, 88% had recurrent glioblastoma identified on [^18^F]FPPRGD_2_ PET, whereas [^18^F]FDG PET enabled identification of recurrence in 76% of patients. In one patient (6%) with recurrent glioblastoma, abnormalities were detected only on [^18^F]FPPRGD_2_ PET, whereas all other patients also showed abnormalities on MRI. Two patients (12%) had no signs of recurrent glioblastoma on PET or MRI [19].

None of the included studies reported on adverse events after the injection of radiotracer for either PET-CT or SPECT-CT imaging [12, 14-20], although this was specifically mentioned as a secondary outcome in only three studies [12, 18, 19].

Quality assessment of the papers revealed that for most elements, the risk of bias of the included studies was determined to be low (**Table 3**). It was unclear for four of the five studies that used a reference standard (i.e., [^18^F]FDG PET) whether the index test and reference standard were assessed in a blinded setting. Only the article of Iagaru et al. detailed that the index test (i.e., [^18^F]FPPRGD_2_ PET-CT) outcomes were assessed without knowledge of the outcome of the reference standard (i.e., [^18^F]FDG PET-CT images and brain MRI data) and vice versa [19].

INSERT TABLE 3 HERE

# Discussion

This systematic review reports on the advantages and challenges of molecular imaging techniques aimed at angiogenesis in neuro-oncological disease by use of radiolabeled RGD peptides. Despite substantial differences in imaging protocols, overall it can be concluded that RGD-targeted PET-CT and SPECT-CT imaging targeting the αvβ_3_ integrin subtype demonstrates superiority over [^18^F]FDG PET-CT in detecting primary and secondary brain tumor lesions due to its higher TBR values, and shows potential for predicting treatment response and post-therapeutic evaluation. One study reported a strong correlation between tracer binding (evaluated with SUVmax) and integrin expression after histopathological evaluation. None of the studies reported adverse events, underscoring its clinical applicability. The heterogeneity in used RGD-based tracers might, at least in part, explain the difference in tracer accumulation between studies. It has been shown that the linker group has little impact on the αvβ_3_ binding affinity of cyclic RGD dimers. The αvβ_3_ binding affinity of NOTA-4P-RGD3 was found to be almost identical to that of NOTA-Galacto-RGD2 and NOTA-I2P-RGD2, despite the differences in peptide multiplicity. The αvβ_3_ binding affinity of DOTA-3P-RGK2, on the other hand, was found to be at least twenty times lower than those of NOTA-Galacto-RGD2, NOTA-I2P-RGD2, NOTA-4P-RGD3, suggesting that αvβ_3_-binding of these RGD-compounds is highly specific [21].

## Specific binding vs. non-specific accumulation

A variety of RGD-based tracers targeting the αvβ_3_ integrin subtype have been reviewed in this study and the majority of studies showed a high tracer accumulation within gliomas, brain metastases and meningioma. Since RGD-radiolabeled tracers cannot cross the intact blood-brain barrier [12], it can be assumed that tracer accumulation is at least in part the result of non-specific accumulation due to more pronounced leakage of the tracer. This leakage can be caused by the severely disintegrated blood-brain barrier in more aggressive gliomas or may be the result from leaking tumor micro-vessels. However, in the study of Li et al. [16], it can be appreciated that regions with contrast-enhancement on MRI do not necessarily overlap with regions of high tracer accumulation. Since contrast-enhancement is a marker of blood-brain barrier disruption, this observation is an argument against solely non-specific leakage of the tracer. Furthermore, another study by Li et al. observed only a moderate [^68^Ga]NOTA-PRGD2 accumulation in high grade gliomas [15]. This also forms an argument against non-specific tracer accumulation. Immunohistochemical αvβ_3_ integrin expression of corresponding tumor samples was found to be highly correlated with the level of tracer uptake according to the study of Schnell et al. [20]. Unfortunately, no other studies are known that performed histopathological validation of the αvβ_3_ integrin expression and tracer uptake. Future studies could help to solve the question with regard to the binding potential of RGD-based tracers to the αvβ_3_ integrin subtype in adult diffuse glioma. For example, a dual-tracer imaging protocol study could help to discern specific from non-specific binding. On the first day, a perfusion PET-tracer (e.g., [^15^O]H_2_O, [^13^N]Ammonia, and ^82^Rb) is administered to assess perfusion and leakage of the tracer through the disintegrated blood brain barrier. The second PET-assessment will be carried out using a PET-tracer targeting αvβ_3_ integrin. By comparing these data within-subject, the component of specific vs. non-specific binding will become apparent.

## Alternative PET agents targeting angiogenesis

The current review focuses on RGD-based imaging tracers for the visualization of angiogenesis, a process much needed for neuro-oncological lesions to provide nutrients and other building blocks [22]. Tumor cells facilitate this by initiating and upregulating angiogenesis, significantly increasing the number of vessels supplying the tumor. Angiogenesis is, however, a complex process in which tissue cells and their surrounding stroma interact and produce growth factors like vascular endothelial growth factor (VEGF) which attract and stimulate endothelial and mesenchymal cells to form new (micro)vessels [23-25].

Next to radiolabeled RGD-peptides aimed at the integrins, a variety of other PET tracers have been developed to target angiogenesis and newly formed (micro)vessels. [^68^Ga]PSMA, [^18^F]DCFPyL and [^89^Zr]Df-IAB2M have been described to bind to the prostate-specific membrane antigen (PSMA). PSMA, which is less well-known as glutamate carboxypeptidase 2 is a receptor that is believed to induce angiogenesis in pathological conditions like tumors independently from the presence of VEGF. PSMA is variably expressed on newly formed blood vessels in tumors, while it is not expressed on healthy brain parenchymal cells or normal vessels. As transport over the blood-brain barrier is impossible, the blood-brain barrier needs to be disintegrated and by consequence, radiotracer accumulation depends on the tumor type [26]. This is similar to radiolabeled RGD-peptides aimed at the integrins. [^68^Ga]PSMA is used most often because of its wide availability for prostate cancer imaging. [^18^F]DCFPyL and other [^18^F]-coupled radiotracers have similar biological properties, though [^89^Zr]Df-IAB2M is a small part of the PSMA antibody and shows faster clearance, thereby achieving higher TBR values as compared to the other two agents [27]. Although preliminary work showed high TBR values in glioblastoma patients [28-30], a recent multicenter study in which different PSMA tracers were applied showed that there was no correlation between tracer uptake and PSMA receptor density on tumor cells of microvessels [31]. This forms a strong argument that PSMA tracers show non-specific accumulation in adult diffuse glioma and represent increased permeability of the blood brain barrier, in combination with leaky tumor microvessels [24, 25]. This hypothesis is strengthened by reports on high uptake in enhancing radiation necrosis and ischemia [32, 33].

Another promising strategy for imaging tumor angiogenesis concerns the targeting of VEGF. VEGFs are a family of mitogenic glycoproteins that promote angiogenesis by the activation of the VEGF receptor via a tyrosine kinase signaling pathway [34]. ^64^Cu-1,4,7-triazacyclononane-1,4,7-triacetic acid-*p*-isothiocyanatobenzyl-bevacizumab-IRDye 800CW (^64^Cu-NOTA-Bev-800CW), [^89^Zr]bevacizumab [35] and [^89^Zr]ranibizumab [36] have been proposed as PET tracers to study human VEGF levels. In one study, it has been described that the [^89^Zr]ranibizumab-PET signal is the sum of perfusion of the tracer into the tumor followed by binding to VEGF, and therefore a resultant of changed perfusion, mean vessel density and VEGF expression, reflecting VEGF biodistribution and bioavailability and allowing in vivo insight in overall tumor angiogenesis [36].

## Possible impact in the clinical setting

In the setting of discerning recurrence from treatment-related abnormalities in post-treatment glioblastoma patients, the Response Assessment in Neuro-Oncology working group has recommended the use of PET imaging [37]. A variety of PET tracers are available for this purpose and recent meta-analyses investigated the diagnostic accuracy of each. O-(2-[^18^F]fluoroethyl)-L-tyrosine ([^18^F]FET) and [S-methyl-^11^C]methionine ([^11^C]MET) were reportedly the most accurate radiotracers for this clinical purpose [38, 39]. However, the works of Schnell et al. [20] and Iagaru et al. [19] indicate that RGD-based PET imaging could be a valuable diagnostic tool for this purpose as well. More research is needed as there are no prospective studies available on this topic. When considering future research directions for the clinical application of RGD-targeted PET imaging, we recommend that researchers prioritize the integration of molecular imaging findings into personalized treatment strategies. This approach has the potential to enhance patient outcomes by tailoring therapies to the unique biological characteristics of individual tumors. Such advancements could facilitate broader utilization in diverse medical contexts, further solidifying the role of RGD-targeted PET imaging in modern precision medicine for neuro-oncology patients. However, as mentioned before, the binding specificity of RGD-tracers should be investigated first. Another relevant topic prior to clinical implementation concerns the large variation in SUVmax values described by Yu et al. [12]. The authors hypothesized that this large variation probably reflects differences in integrin expression across lesions of different origins and variability in αvβ_3_ expression among cancer patients. For that reason, within subject-normalization could be considered in the clinical setting.

# Conclusion

This review shows that RGD-targeted PET and SPECT imaging could be a promising tool for neuro-oncology as it provides new molecular information on the lesions. However, the specificity of RGD-tracers remains partially elusive and should therefore be studied further before RGD-targeted PET can be implemented in clinical practice. Radiopharmacodynamic, -kinetic and modelling studies in humans are much needed to bring this type of tracer further from bench to bedside in patients with neurooncological disorders.

# References

1. Folkman J. Angiogenesis in cancer, vascular, rheumatoid and other disease. Nat Med. 1995;1:27-31. doi:10.1038/nm0195-27.

2. Cao Y, Arbiser J, D'Amato RJ, D'Amore PA, Ingber DE, Kerbel R, et al. Forty-year journey of angiogenesis translational research. Sci Transl Med. 2011;3:114rv3. doi:10.1126/scitranslmed.3003149.

3. Plow EF, Haas TA, Zhang L, Loftus J, Smith JW. Ligand binding to integrins. J Biol Chem. 2000;275:21785-8. doi:10.1074/jbc.R000003200.

4. Hynes RO. Integrins: bidirectional, allosteric signaling machines. Cell. 2002;110:673-87. doi:10.1016/s0092-8674(02)00971-6.

5. Xiao L, Xin J. Advances in Clinical Oncology Research on (99m)Tc-3PRGD2 SPECT Imaging. Front Oncol. 2022;12:898764. doi:10.3389/fonc.2022.898764.

6. Debordeaux F, Chansel-Debordeaux L, Pinaquy JB, Fernandez P, Schulz J. What about alpha(v)beta(3) integrins in molecular imaging in oncology? Nucl Med Biol. 2018;62-63:31-46. doi:10.1016/j.nucmedbio.2018.04.006.

7. Louis DN, Perry A, Wesseling P, Brat DJ, Cree IA, Figarella-Branger D, et al. The 2021 WHO Classification of Tumors of the Central Nervous System: a summary. Neuro Oncol. 2021;23:1231-51. doi:10.1093/neuonc/noab106.

8. Price M, Ballard C, Benedetti J, Neff C, Cioffi G, Waite KA, et al. CBTRUS Statistical Report: Primary Brain and Other Central Nervous System Tumors Diagnosed in the United States in 2017-2021. Neuro Oncol. 2024;26:vi1-vi85. doi:10.1093/neuonc/noae145.

9. Hall WA, Djalilian HR, Nussbaum ES, Cho KH. Long-term survival with metastatic cancer to the brain. Med Oncol. 2000;17:279-86. doi:10.1007/BF02782192.

10. Long YY, Chen J, Xie Y, Wang Y, Wu YZ, Xv Y, et al. Long-term survival with a combination of immunotherapy, anti-angiogenesis, and traditional radiotherapy in brain metastatic small cell lung cancer: a case report. Front Oncol. 2023;13:1209758. doi:10.3389/fonc.2023.1209758.

11. Salari K, Lee JS, Ye H, Seymour ZA, Lee KC, Chinnaiyan P, et al. Long-term survival in patients with brain-only metastatic non-small cell lung cancer undergoing upfront intracranial stereotactic radiosurgery and definitive treatment to the thoracic primary site. Radiother Oncol. 2024;196:110262. doi:10.1016/j.radonc.2024.110262.

12. Yu C, Pan D, Mi B, Xu Y, Lang L, Niu G, et al. (18)F-Alfatide II PET/CT in healthy human volunteers and patients with brain metastases. Eur J Nucl Med Mol Imaging. 2015;42:2021-8. doi:10.1007/s00259-015-3118-2.

13. Whiting PF, Rutjes AW, Westwood ME, Mallett S, Deeks JJ, Reitsma JB, et al. QUADAS-2: a revised tool for the quality assessment of diagnostic accuracy studies. Ann Intern Med. 2011;155:529-36. doi:10.7326/0003-4819-155-8-201110180-00009.

14. Kang F, Wang S, Tian F, Zhao M, Zhang M, Wang Z, et al. Comparing the Diagnostic Potential of 68Ga-Alfatide II and 18F-FDG in Differentiating Between Non-Small Cell Lung Cancer and Tuberculosis. J Nucl Med. 2016;57:672-7. doi:10.2967/jnumed.115.167924.

15. Li D, Zhang J, Ji N, Zhao X, Zheng K, Qiao Z, et al. Combined 68Ga-NOTA-PRGD2 and 18F-FDG PET/CT Can Discriminate Uncommon Meningioma Mimicking High-Grade Glioma. Clin Nucl Med. 2018;43:648-54. doi:10.1097/RLU.0000000000002233.

16. Li D, Zhao X, Zhang L, Li F, Ji N, Gao Z, et al. (68)Ga-PRGD2 PET/CT in the evaluation of Glioma: a prospective study. Mol Pharm. 2014;11:3923-9. doi:10.1021/mp5003224.

17. Zhang H, Liu N, Gao S, Hu X, Zhao W, Tao R, et al. Can an (1)(8)F-ALF-NOTA-PRGD2 PET/CT Scan Predict Treatment Sensitivity to Concurrent Chemoradiotherapy in Patients with Newly Diagnosed Glioblastoma? J Nucl Med. 2016;57:524-9. doi:10.2967/jnumed.115.165514.

18. Song YS, Park HS, Lee BC, Jung JH, Lee HY, Kim SE. Imaging of Integrin alpha(v)beta(3) Expression in Lung Cancers and Brain Tumors Using Single-Photon Emission Computed Tomography with a Novel Radiotracer (99m)Tc-IDA-D-[c(RGDfK)](2). Cancer Biother Radiopharm. 2017;32:288-96. doi:10.1089/cbr.2017.2233.

19. Iagaru A, Mosci C, Mittra E, Zaharchuk G, Fischbein N, Harsh G, et al. Glioblastoma Multiforme Recurrence: An Exploratory Study of (18)F FPPRGD2 PET/CT. Radiology. 2015;277:497-506. doi:10.1148/radiol.2015141550.

20. Schnell O, Krebs B, Carlsen J, Miederer I, Goetz C, Goldbrunner RH, et al. Imaging of integrin alpha(v)beta(3) expression in patients with malignant glioma by [18F] Galacto-RGD positron emission tomography. Neuro Oncol. 2009;11:861-70. doi:10.1215/15228517-2009-024.

21. Zhao ZQ, Ji S, Li XY, Fang W, Liu S. (68)Ga-labeled dimeric and trimeric cyclic RGD peptides as potential PET radiotracers for imaging gliomas. Appl Radiat Isot. 2019;148:168-77. doi:10.1016/j.apradiso.2019.03.033.

22. Hardee ME, Zagzag D. Mechanisms of glioma-associated neovascularization. The American journal of pathology. 2012;181:1126-41. doi:10.1016/j.ajpath.2012.06.030.

23. Colman HA, K. Molecular Pathogenesis. In: P. NARDW, editor. Primary Central Nervous System Tumors Current Clinical Oncology: Humana Press; 2011. p. 27-44.

24. Nussenbaum F, Herman IM. Tumor angiogenesis: insights and innovations. J Oncol. 2010;2010:132641. doi:10.1155/2010/132641.

25. De Palma M, Biziato D, Petrova TV. Microenvironmental regulation of tumour angiogenesis. Nat Rev Cancer. 2017;17:457-74. doi:10.1038/nrc.2017.51.

26. Nomura N, Pastorino S, Jiang P, Lambert G, Crawford JR, Gymnopoulos M, et al. Prostate specific membrane antigen (PSMA) expression in primary gliomas and breast cancer brain metastases. Cancer cell international. 2014;14:26. doi:10.1186/1475-2867-14-26.

27. Matsuda M, Ishikawa E, Yamamoto T, Hatano K, Joraku A, Iizumi Y, et al. Potential use of prostate specific membrane antigen (PSMA) for detecting the tumor neovasculature of brain tumors by PET imaging with (89)Zr-Df-IAB2M anti-PSMA minibody. Journal of neuro-oncology. 2018. doi:10.1007/s11060-018-2825-5.

28. Brighi C, Puttick S, Woods A, Keall P, Tooney PA, Waddington DEJ, et al. Comparison between [(68)Ga]Ga-PSMA-617 and [(18)F]FET PET as Imaging Biomarkers in Adult Recurrent Glioblastoma. Int J Mol Sci. 2023;24. doi:10.3390/ijms242216208.

29. Kunikowska J, Bartosz K, Leszek K. Glioblastoma multiforme: another potential application for (68)Ga-PSMA PET/CT as a guide for targeted therapy. Eur J Nucl Med Mol Imaging. 2018;45:886-7. doi:10.1007/s00259-018-3934-2.

30. Kunikowska J, Charzynska I, Kulinski R, Pawlak D, Maurin M, Krolicki L. Tumor uptake in glioblastoma multiforme after IV injection of [(177)Lu]Lu-PSMA-617. Eur J Nucl Med Mol Imaging. 2020;47:1605-6. doi:10.1007/s00259-020-04715-z.

31. van Lith SAM, Pruis IJ, Tolboom N, Snijders TJ, Henssen D, Ter Laan M, et al. PET Imaging and Protein Expression of Prostate-Specific Membrane Antigen in Glioblastoma: A Multicenter Inventory Study. J Nucl Med. 2023;64:1526-31. doi:10.2967/jnumed.123.265738.

32. Sasikumar A, Joy A, Pillai MR, Nanabala R, Anees KM, Jayaprakash PG, et al. Diagnostic Value of 68Ga PSMA-11 PET/CT Imaging of Brain Tumors-Preliminary Analysis. Clinical nuclear medicine. 2017;42:e41-e8. doi:10.1097/rlu.0000000000001451.

33. Salas Fragomeni RA, Pienta KJ, Pomper MG, Gorin MA, Rowe SP. Uptake of Prostate-Specific Membrane Antigen-Targeted 18F-DCFPyL in Cerebral Radionecrosis: Implications for Diagnostic Imaging of High-Grade Gliomas. Clinical nuclear medicine. 2018;43:e419-e21. doi:10.1097/rlu.0000000000002280.

34. Jubb AM, Harris AL. Biomarkers to predict the clinical efficacy of bevacizumab in cancer. Lancet Oncol. 2010;11:1172-83. doi:10.1016/S1470-2045(10)70232-1.

35. Nagengast WB, de Vries EG, Hospers GA, Mulder NH, de Jong JR, Hollema H, et al. In vivo VEGF imaging with radiolabeled bevacizumab in a human ovarian tumor xenograft. J Nucl Med. 2007;48:1313-9. doi:10.2967/jnumed.107.041301.

36. Nagengast WB, Lub-de Hooge MN, Oosting SF, den Dunnen WF, Warnders FJ, Brouwers AH, et al. VEGF-PET imaging is a noninvasive biomarker showing differential changes in the tumor during sunitinib treatment. Cancer Res. 2011;71:143-53. doi:10.1158/0008-5472.CAN-10-1088.

37. Albert NL, Weller M, Suchorska B, Galldiks N, Soffietti R, Kim MM, et al. Response Assessment in Neuro-Oncology working group and European Association for Neuro-Oncology recommendations for the clinical use of PET imaging in gliomas. Neuro Oncol. 2016;18:1199-208. doi:10.1093/neuonc/now058.

38. de Zwart PL, van Dijken BRJ, Holtman GA, Stormezand GN, Dierckx R, Jan van Laar P, et al. Diagnostic Accuracy of PET Tracers for the Differentiation of Tumor Progression from Treatment-Related Changes in High-Grade Glioma: A Systematic Review and Metaanalysis. J Nucl Med. 2020;61:498-504. doi:10.2967/jnumed.119.233809.

39. Henssen D, Leijten L, Meijer FJA, van der Kolk A, Arens AIJ, Ter Laan M, et al. Head-To-Head Comparison of PET and Perfusion Weighted MRI Techniques to Distinguish Treatment Related Abnormalities from Tumor Progression in Glioma. Cancers (Basel). 2023;15. doi:10.3390/cancers15092631.

Declarations

## *Ethics approval*

Ethical approval was not applicable since this concerns a systematic review. Clinical trial number not applicable.

## *Consent to participate*

Informed consent was not applicable since this concerns a systematic review.

## *Author contributions*

Conceptualization— D.H., S.H., A.A., A.K.; Methodology— D.H, S.H.; Validation— A.A., A.K., O.S., Sw.H., M.G.; Formal analysis— D.H., S.H.; Investigation— D.H., S.H.; Resources— D.H., A.A., O.S., S.H., M.G.; Data curation— D.H., S.H.; Writing (original draft preparation) — D.H., S.H. Writing (review and editing) — A.A., A.K., O.S., Sw.H., M.G.; Visualization/Table construction — D.H., S.H. Supervision — A.A., A.K., O.S., Sw.H., M.G.; Project administration — D.H.; All authors have read and agreed to the published version of the manuscript

## *Competing interests*

The authors declare that no conflicts of interest exist.

## *Funding*

No funding was received for this study.

## *Data availability*

No specific datasets were generated during the current study; all required information on the origin of the data of this review is made available from this manuscript.

## *Acknowledgements*

Dr. Henssen is supported by the Clinician Scientist Programme of Universitätsmedizin Leipzig, which facilitates the integration of clinical practice and scientific research.
