# Supplementary material for: Advances in clinical neuro-oncology research on integrin PET imaging
Source: EJNMMI Rep. 2025 Sep 29;9(1):33. doi: 10.1186/s41824-025-00270-8 (PMC12477096; doi:10.1186/s41824-025-00270-8)
Supplement: Supplementary file 2 — Supplementary Material 2 [file 41824_2025_270_MOESM2_ESM.docx]

Dylan Henssen, MD PhD

Universitätsklinikum Leipzig

Department of Nuclear Medicine

Nuclear Medicine Physician & Radiologist

[Dylan.henssen@medizin.uni-leipzig.de](mailto:Dylan.henssen@medizin.uni-leipzig.de)

Radboudumc

Department of Medical Imaging

Researcher Molecular Imaging and Radiology

[Dylan.henssen@radboudumc.nl](mailto:Dylan.henssen@radboudumc.nl)

Deputy Editor, EJNMMI Reports

Editor-in-Chief, EJNMMI Reports

**Date:** 2025-08-14

**Subject:** Reply to the editor and reviewers concerning “*EJHI-D-25-00040 Advances in clinical neuro-oncology research on integrin PET imaging Dylan Henssen; Siem Herings; Osama Sabri; Swen Hesse; Anja van der Kolk; Anne I.J. Arens; Martin Gotthardt EJNMMI Reports”*

Dear editors,

Thank you for your letter and the revisions suggested by your reviewers. The insightful commentaries helped us to further improve the manuscript. We would like to submit a revised version of our manuscript to Brain Research Bulletin. Please find attached the response to the reviewers’ comments as well as the revised version of the manuscript. The changes made to manuscript are highlighted by track-changes.

We hope that this letter, combined with the revised manuscript provides answers to the questions of the reviewers in a sufficient manner.

If anything remains unclear, please do not hesitate to contact me at: [dylan.henssen@radboudumc.nl](mailto:dylan.henssen@radboudumc.nl).

With kind regards, also on behalf of the co-authors,

Dylan J.H.A. Henssen, M.D. Ph.D.

EJHI-D-25-00040

Advances in clinical neuro-oncology research on integrin PET imaging Dylan Henssen; Siem Herings; Osama Sabri; Swen Hesse; Anja van der Kolk; Anne I.J. Arens; Martin Gotthardt EJNMMI Reports

Dear Dr Henssen,

Your manuscript 'Advances in clinical neuro-oncology research on integrin PET imaging' (EJHI-D-25-00040) has been assessed by our reviewers. Based on these reports, and my own assessment as Editor, I am pleased to inform you that it is potentially acceptable for publication in EJNMMI Reports, once you have carried out some essential revisions suggested by our reviewers.

Their reports, together with any other comments, are below. Please also take a moment to check our website at <https://www.editorialmanager.com/ejhi/> for any additional comments that were saved as attachments.

Once you have made the necessary corrections, please submit a revised manuscript online at:

<https://www.editorialmanager.com/ejhi/>

If you have forgotten your username or password please use the 'Send Login Details' link to get your login information. For security reasons, your password will be reset.

Please include a point-by-point response within the 'Response to Reviewers' box in the submission system and highlight (with 'tracked changes'/coloured/underlines/highlighted text) all changes made when revising the manuscript. Please ensure you describe additional experiments that were carried out and include a detailed rebuttal of any criticisms or requested revisions that you disagreed with. Please also ensure that your revised manuscript conforms to the journal style, which can be found in the Submission Guidelines on the journal homepage.

The due date for submitting the revised version of your article is 21 Aug 2025

Please note: When uploading your revised manuscript files, please make sure only to submit your editable source files (i.e. Word, Tex). PDF is not allowed at this stage.

Please note, if your manuscript is accepted you will not be able to make any changes to the authors, or order of authors, of your manuscript once the editor has accepted your manuscript for publication. If you wish to make any changes to authorship before you resubmit your revisions, please reply to this email and ask for a 'Request for change in authorship' form which should be completed by all authors (including those to be removed) and returned to this email address. Please ensure that any changes in authorship fulfil the criteria for authorship as outlined in SpringerOpen's editorial policies (<http://www.springeropen.com/get-published/editorial-policies#authorship>).

Once you have completed and returned the form, your request will be considered and you will be advised whether the requested changes will be allowed.

By resubmitting your manuscript you confirm that all author details on the revised version are correct, that all authors have agreed to authorship and order of authorship for this manuscript and that all authors have the appropriate permissions and rights to the reported data.

Please be aware that we may investigate, or ask your institute to investigate, any unauthorised attempts to change authorship or discrepancies in authorship between the submitted and revised versions of your manuscript.

We look forward to receiving your revised manuscript soon.

Best wishes,

Federico Caobelli

EJNMMI Reports

<http://ejhi.springeropen.com/>

Reviewer reports:

Reviewer #1:

The manuscript provides a comprehensive and well-structured review of current evidence on the use of RGD-based tracers for PET imaging in neuro-oncology, with a particular focus on diagnostic performance and treatment response evaluation. The authors effectively synthesize the literature, presenting both the clinical utility and the limitations of RGD-based tracers. The paper is well written, with clear language, an informative introduction, transparent methodology, well-organized results and an insightful discussion. The authors also identify gaps in current knowledge and propose meaningful directions for future research.

We thank the reviewer for the meticulous reading of our manuscript.

The following minor typographical errors were noted:

-	Introduction / Lines 10-11: typo (comma can be removed) "Although angiogenesis, is a highly orchestrated…"

Typographical error was corrected.

-	Materials and Methods (Quality assessment) / Lines 5-8: could be rephrased as follows "This checklist is used to assess the risk of bias and concerns regarding applicability in primary diagnostic accuracy studies."

This was rephrased as suggested by the reviewer. The section now reads:

*“The methodological quality of the included articles was assessed using the QUADAS-2 checklist [13]. This checklist was used to assess the risk of bias and concerns regarding applicability in primary diagnostic accuracy studies. Two investigators (D.H. and S.H.) independently assessed the methodological quality of the included articles. Any discrepancies were resolved by discussion. “*

-	Results / Page 7 Line 20: typo ("systemic" -> "sistematic")

Typographical error was corrected.

-	Results / Page 9 Lines 7-12: could be rephrased as follows "When a SUVmax threshold of 1.35 was applied, the sensitivity, specificity, and accuracy were 83.3%, 88.9%, and 85.7%, respectively."

Rephrasing was carried out accordingly.

-	Results / Page 9 Line 29: typo (missing preposition) "… recurrence in 76% of patients"

Typographical error was corrected.

-	Results / Page 9 Lines 29-34: could be rephrased as follows "In one patient (6%) with recurrent glioblastoma, abnormalities were detected only on [18F]FPPRGD2 PET, whereas all other patients also showed abnormalities on MRI."

Rephrasing was carried out accordingly.

Finally, given the systematic nature of this literature review, the methodological transparency could be enhanced by reporting the exact PubMed and Embase search strings in the Supplementary Materials (the Materials and Methods section currently provides only a brief overview of the main search terms).

We agree with the reviewer that this would be beneficial for the transparency of our manuscript. This information is now provided in Table 1 in the revised version of our manuscript. For your convenience, the revised Table 1 is also printed below.

| *Database* | *Search String* |
| --- | --- |
| PubMed | ((("Brain Neoplasms"[Mesh]) OR (((brain metasta*[Title/Abstract]) OR ("Brain Neoplasms/secondary"[Mesh]) OR (((glioma[Title/Abstract]) OR (glioblastoma[Title/Abstract])) OR ((("Glioma"[Mesh]) OR "Glioma/diagnostic imaging"[Mesh]) OR (("Glioblastoma"[Mesh]) OR "Glioblastoma/diagnostic imaging"[Mesh]))))) AND ((((((Positron Emission Tomography Computed Tomography[Title/Abstract]) OR (PET/CT[Title/Abstract])) OR (PET-CT[Title/Abstract])) OR (("Positron Emission Tomography Computed Tomography"[MAJR]) OR "Positron Emission Tomography Computed Tomography"[MAJR])) AND (((("Integrin alphaVbeta3/metabolism"[MeSH]) OR "Endothelial Cells/metabolism"[MeSH]) OR ("Integrin alphaVbeta3"[nm])) OR (Integrin alphaVbeta3[Title/Abstract]))))) |
| EMBASE | ((Integrin alphaVbeta3.mp. or exp vitronectin receptor/) AND (((glioma or glioblastoma).mp. [mp=title, abstract, heading word, drug trade name, original title, device manufacturer, drug manufacturer, device trade name, keyword heading word, floating subheading word, candidate term word])) OR ((brain metastasis or brain meta*).mp. [mp=title, abstract, heading word, drug trade name, original title, device manufacturer, drug manufacturer, device trade name, keyword heading word, floating subheading word, candidate term word]))) OR ((brain neoplasm*).mp. [mp=title, abstract, heading word, drug trade name, original title, device manufacturer, drug manufacturer, device trade name, keyword heading word, floating subheading word, candidate term word]))) AND ((Positron Emission Tomography Computed Tomography or PET CT).mp. [mp=title, abstract, heading word, drug trade name, original title, device manufacturer, drug manufacturer, device trade name, keyword heading word, floating subheading word, candidate term word])) |
| Cochrane Library | (brain tumor or brain metastas* or glioma or glioblastoma) AND (positron emission tomography or PET or PET CT or Positron Emission Tomography Computed Tomography) AND (integrin alpha V beta3 or integrin) |

**Table 1 Search string per database**

Reviewer #2:

The manuscript is focused on RGD-based tracers for PET imaging in neuro-oncology. The authors deeply discussed potential clinical use and the limitations of these new tracers. Unmet needs and future perspectives are also addressed.
We thank the reviewer for the meticulous reading of our manuscript.

As this is a systematic review, I believe it is necessary to specify which search strings were used within the datasets considered (PubMed and Embase...). Few methodological info are presently provided in the methods.

The complete search strings are now provided in the revised manuscript, as suggested by the reviewer. We refer to Table 1, which is also printed below for your convenience. Furthermore, we have revised the Materials and Methods section following the suggestions of the reviewers, which also allowed us to elaborate on some methodological steps.

| *Database* | *Search String* |
| --- | --- |
| PubMed | ((("Brain Neoplasms"[Mesh]) OR (((brain metasta*[Title/Abstract]) OR ("Brain Neoplasms/secondary"[Mesh]) OR (((glioma[Title/Abstract]) OR (glioblastoma[Title/Abstract])) OR ((("Glioma"[Mesh]) OR "Glioma/diagnostic imaging"[Mesh]) OR (("Glioblastoma"[Mesh]) OR "Glioblastoma/diagnostic imaging"[Mesh]))))) AND ((((((Positron Emission Tomography Computed Tomography[Title/Abstract]) OR (PET/CT[Title/Abstract])) OR (PET-CT[Title/Abstract])) OR (("Positron Emission Tomography Computed Tomography"[MAJR]) OR "Positron Emission Tomography Computed Tomography"[MAJR])) AND (((("Integrin alphaVbeta3/metabolism"[MeSH]) OR "Endothelial Cells/metabolism"[MeSH]) OR ("Integrin alphaVbeta3"[nm])) OR (Integrin alphaVbeta3[Title/Abstract]))))) |
| EMBASE | ((Integrin alphaVbeta3.mp. or exp vitronectin receptor/) AND (((glioma or glioblastoma).mp. [mp=title, abstract, heading word, drug trade name, original title, device manufacturer, drug manufacturer, device trade name, keyword heading word, floating subheading word, candidate term word])) OR ((brain metastasis or brain meta*).mp. [mp=title, abstract, heading word, drug trade name, original title, device manufacturer, drug manufacturer, device trade name, keyword heading word, floating subheading word, candidate term word]))) OR ((brain neoplasm*).mp. [mp=title, abstract, heading word, drug trade name, original title, device manufacturer, drug manufacturer, device trade name, keyword heading word, floating subheading word, candidate term word]))) AND ((Positron Emission Tomography Computed Tomography or PET CT).mp. [mp=title, abstract, heading word, drug trade name, original title, device manufacturer, drug manufacturer, device trade name, keyword heading word, floating subheading word, candidate term word])) |
| Cochrane Library | (brain tumor or brain metastas* or glioma or glioblastoma) AND (positron emission tomography or PET or PET CT or Positron Emission Tomography Computed Tomography) AND (integrin alpha V beta3 or integrin) |

**Table 1 Search string per database**

Furthermore, the revised Materials and Methods section now reads:

*“Literature search and screening*

*A systematic literature search was performed in PubMed, EMBASE and the Cochrane Library. The search terms included the following: “Glioblastoma”, “Glioma”, "Brain Neoplasms/primary" “Brain metastasis”, "Brain Neoplasms/secondary", “Positron Emission Tomography Computed Tomography”, "Integrin alphaVbeta3", "Endothelial Cells”, “Endothelial Cells Metabolism". The three databases were consulted until November 2024 by a single investigator (D.H.). The complete search string is provided in* ***Table 1****.*

*INSERT TABLE 1 HERE*

*The retrieved articles were imported into Rayyan (*[*www.rayyan.ai*](http://www.rayyan.ai)*), a systematic review tool that aids in the blinded screening of publications and detects duplicate findings. After processing the duplicate findings, the remaining titles and abstracts were screened independently by two researchers (D.H., S.H.) blinded to each other’s decisions. During this screening the researchers determined which papers should be included and which should be excluded based on the abstract. Incongruently assessed papers were discussed after which a consensus decision was made. This procedure was followed by a full-text screening of the retrieved articles, utilizing the same setup.*

*Extracted data and synthesis of results*

*The papers included after full-text assessment were analyzed by two investigators independently (D.H. and S.H.). Predefined, standard data-extraction sheets were used and the following data were extracted: I) Demographic data of studied population, II) type of tumors included, III) number of tumors, IV) utilized radiotracer, V) dose administered in MBq, VI) targeted receptor (integrin subtype), VII) scan time post-injection, VIII) major findings, and IX) limitations of the study. A comprehensive overview of the current state of integrin-targeting tracers in neuro-oncology is provided by use of a narrative description of patients, imaging intervention, primary outcome and main limitations of the study.*

*Quality assessment*

*The methodological quality of the included articles was assessed using the QUADAS-2 checklist [13]. This checklist was used to assess the risk of bias and concerns regarding applicability in primary diagnostic accuracy studies. Two investigators (D.H. and S.H.) independently assessed the methodological quality of the included articles. Any discrepancies were resolved by discussion. “*
